# Uropathogenic *Escherichia coli* P and Type 1 Fimbriae Act in Synergy in a Living Host to Facilitate Renal Colonization Leading to Nephron Obstruction

**DOI:** 10.1371/journal.ppat.1001298

**Published:** 2011-02-24

**Authors:** Keira Melican, Ruben M. Sandoval, Abdul Kader, Lina Josefsson, George A. Tanner, Bruce A. Molitoris, Agneta Richter-Dahlfors

**Affiliations:** 1 Department of Neuroscience, Swedish Medical Nanoscience Center, Karolinska Institutet, Stockholm, Sweden; 2 Division of Nephrology, Department of Medicine, Indiana Center for Biological Microscopy, Indiana University School of Medicine, Indianapolis, Indiana, United States of America; 3 Department of Cellular and Integrative Physiology, Indiana University School of Medicine, Indianapolis, Indiana, United States of America; Yale University School of Medicine, United States of America

## Abstract

The progression of a natural bacterial infection is a dynamic process influenced by the physiological characteristics of the target organ. Recent developments in live animal imaging allow for the study of the dynamic microbe-host interplay in real-time as the infection progresses within an organ of a live host. Here we used multiphoton microscopy-based live animal imaging, combined with advanced surgical procedures, to investigate the role of uropathogenic *Escherichia coli* (UPEC) attachment organelles P and Type 1 fimbriae in renal bacterial infection. A GFP^+^ expressing variant of UPEC strain CFT073 and genetically well-defined isogenic mutants were microinfused into rat glomerulus or proximal tubules. Within 2 h bacteria colonized along the flat squamous epithelium of the Bowman's capsule despite being exposed to the primary filtrate. When facing the challenge of the filtrate flow in the proximal tubule, the P and Type 1 fimbriae appeared to act in synergy to promote colonization. P fimbriae enhanced early colonization of the tubular epithelium, while Type 1 fimbriae mediated colonization of the center of the tubule via a mechanism believed to involve inter-bacterial binding and biofilm formation. The heterogeneous bacterial community within the tubule subsequently affected renal filtration leading to total obstruction of the nephron within 8 h. Our results reveal the importance of physiological factors such as filtration in determining bacterial colonization patterns, and demonstrate that the spatial resolution of an infectious niche can be as small as the center, or periphery, of a tubule lumen. Furthermore, our data show how secondary physiological injuries such as obstruction contribute to the full pathophysiology of pyelonephritis.

## Author Summary

When bacteria such as uropathogenic *Escherichia coli* (UPEC) infect a living kidney, they face numerous physiological challenges such as the flow of urine. Bacteria need to attach themselves to the epithelial linings of the kidney to withstand this flow. In this work we use a live animal imaging model to study how UPEC colonize a living kidney despite the physiological challenges they face. We show that P and Type 1 fimbriae, two of the most well described UPEC adhesion factors, work together to promote successful bacterial colonization. P fimbriae mediate binding between the bacteria and the epithelial cells lining the tubules, while Type 1 appears to play a role in inter-bacterial binding and biofilm formation in the center parts of the lumen. The heterogeneous bacterial community which filled the tubule was subsequently shown to effect nephron filtration and resulted in a total loss of filtrate flow i.e. obstruction. This work demonstrates the interplay between the bacterial and host aspects, indicating how factors such as filtration may affect bacterial adhesion and vice versa. It also highlights the multifactorial basis of kidney infection, demonstrating how physiological injuries such as obstruction may contribute towards the full pathophysiology of pyelonephritis.

## Introduction

Bacteria colonizing the mammalian host face numerous dynamic challenges. In the urinary tract, this is exemplified by the shear stress of urine flow. This stress can vary considerably; in the bladder, the flow changes dramatically upon voiding whereas in the renal tubules more subtle variations occur as the body regulates renal function. Uropathogenic *E. coli* (UPEC), the major causative agent of urinary tract infections (UTI) have evolved mechanisms by which to overcome these challenges. For successful colonization in this hydrodynamically challenging environment, bacterial attachment to the epithelium is essential. For UTI caused by UPEC, major roles have been ascribed to the attachment organelles Type 1 and P fimbriae [1]. While both are considered important [2], [3], [4], [5], their definitive role in the progression of kidney infection, pyelonephritis, *in vivo* has never been clearly defined.

Type 1 and P fimbriae bind to mono-mannose and globoseries glycosphingolipids, respectively [6], [7]. Lack of mono-mannose rich uroplakin on renal epithelia has previously implied a limited role for Type 1 fimbriae in kidney infection, whereas these fimbriae have been strongly linked to many aspects of bladder infection [2]. The P fimbrial operon has been shown to be over-represented in clinical isolates from pyelonephritic patients, yet has not been demonstrated to be essential for disease [8]. Only subtle roles for P fimbriae-mediated adherence have been described in uroepithelial cell culture models [3], and investigations of its role in ascending infection models have yielded inconsistent and often conflicting results [9], [10], [11], [12]. The conflicting data may reflect the limited spatial and temporal resolution in previously used model systems for *in vivo* infections, highlighting the need to address the problem using alternative techniques.

We have recently employed live animal multiphoton microscopy (MPM) to visualize tissue dynamics during renal bacterial infections [13], [14], [15]. These real-time visualization studies of the infection process can be performed under the influence of all physiological factors, including the vascular, nervous, immune and hormonal systems [14], [16], [17], [18]. Previous study of the acute pathogenesis of kidney infection using live imaging revealed a very rapid bacterial colonization process accompanied by major alterations of tissue homeostasis [19]. During the first 3–4 h, local tissue oxygen tension (PO_2_) plummeted to 0 mm Hg, followed by clotting and cessation of blood flow in peritubular capillaries. The ensuing ischemia was established as a local innate immune defense mechanism, protecting the organism from systemic bacterial spread and sepsis [20]. Bacterial containment at the infection site resulted in a focused immune cell infiltration, which after 24 h resulted in bacterial clearance from the injection site and localized tissue edema. The tissue morphology seen at 24 h was comparable to that seen in renal abscesses in ascending infections 4 days post-infection [19].

In the live animal model, spatial-temporal control of the infection is achieved by slowly infusing bacteria into superficial proximal tubules of surgically exposed kidneys in anesthetized rats. The micro-infusion technique and the length of anesthesia are two important factors in the choice of rats in the present experimental model. Rats have previously been used successfully in experimental ascending UTI [21], [22], [23] models and do express the P fimbriae Galα1-4Galβ receptor for UPEC attachment in the kidney [24]. In our previous work immediate visualization of the infection site revealed that only a few bacteria succeed in initially withstanding renal filtrate flow and colonize the tubule epithelium [19]. The attached bacteria multiplied extensively, filling the entire tubule lumen within 4–5 h. This implies that UPEC can, and do, express sufficient adhesion factors to withstand the mechanical stresses *in vivo*. Here, we investigate the bacterial adhesion mechanisms that enable bacteria to withstand the obstacles to early stage kidney colonization, and define previously unknown synergistic functions of P and Type 1 fimbriae under dynamic *in vivo* conditions.

## Results

### Bacteria adhere to and colonize Bowman's capsule

**Figure 1:**
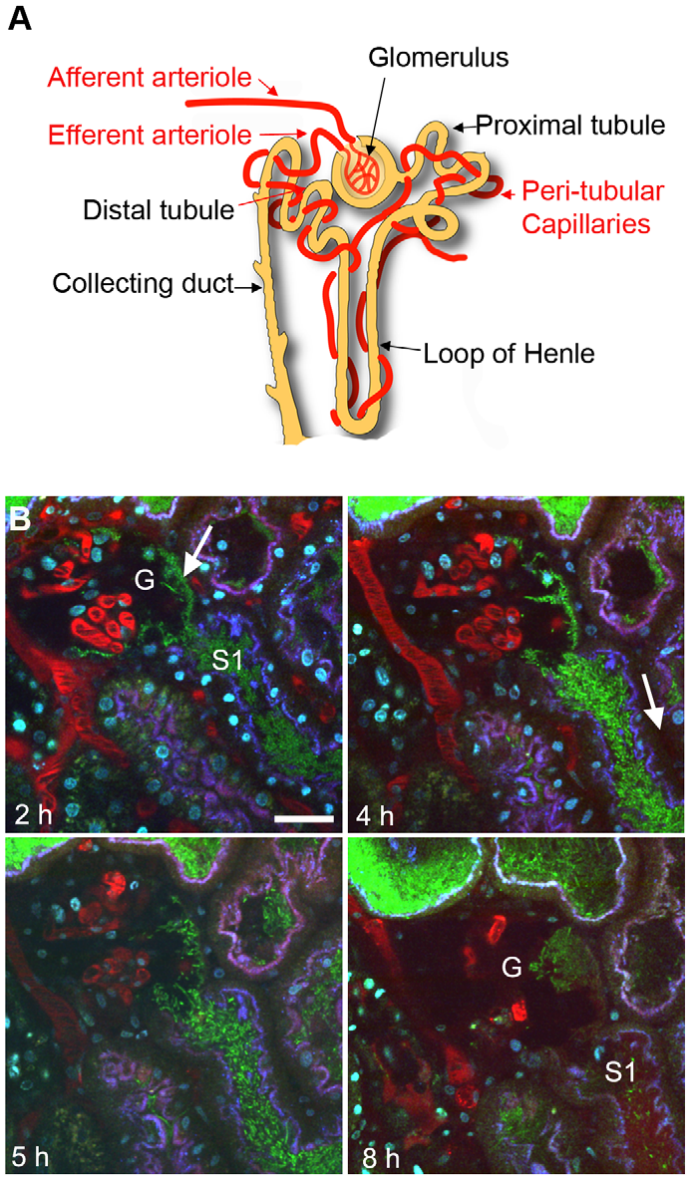
UPEC attachment in Bowman's capsule. (**A**) Schematic showing nephron structure. (**B**) Dynamic imaging of LT004 infused directly into Bowman's capsule. (**2 h**) Bacteria (green, arrow) conformally lining to Bowman's capsule epithelia of a glomerulus (G) and in the early proximal tubule segment (S1). Proximal tubule epithelium is outlined by endocytosed 10 kDa cascade-blue labeled dextran (blue). Blood plasma is labeled with 500 kDa rhodamine dextran (red). Hoechst 33342 labels cell nuclei (cyan). (**4–5 h**) Bacterial colonization within Bowman's capsule and the proximal tubule is accompanied by reduced peritubular capillary blood flow (4 h, arrow). (**8 h**) The glomerular capillary loops succumb to infection and the blood stops flowing (G). Scale bar  = 30 µm.

The initial stages of renal filtration occur in the glomerulus, which consists of capillary tufts surrounded by the Bowman's capsule (Figure 1A). Tissue biopsies from patients with pyelonephritis demonstrate that infection rarely ascend into Bowman's capsule. The disease is therefore characterized as a tubulointerstitial rather than a glomerular disorder [25]. Colonization of the proximal tubule segments of the nephrons is promoted by efficient UPEC attachment to the microvilliated cuboidal epithelia. We hypothesized that the resistance of a functional glomerulus to infection may therefore be due to an inability of bacteria to bind to Bowman's capsule flat squamous epithelium under dynamic conditions. To address this question LT004, a derivative of the prototypic UPEC strain CFT073 [26] expressing GFP^+^ [27] from a single chromosomally inserted gene [19] was slowly infused directly into the Bowman's space of superficial glomeruli in Munich–Wistar rats. As with previous reports of the microinfusion model, multiphoton imaging showed that the vast majority of infused bacteria are immediately flushed out by the filtrate flow, leaving only a few to initiate colonization [19]. Distinct green fluorescence, conformally lining the epithelia of Bowman's capsule, was observed 2 h after infusion, suggestive of bacterial attachment. Extensive multiplication occurred over the following hours, with a mat-like bacterial community being formed both within Bowman's capsule and in the earliest (S1) portion of the proximal tubule (Figure 1B).

Intravenous injection of a fluorescent large molecular weight dextran (a blood plasma marker) revealed that the infection was accompanied by the anticipated decrease [20] in peritubular capillary flow (Figure 1B). The glomerular capillaries and adjacent arterioles however appeared more robust, with blood flow continuing hours after the shutdown of peritubular capillaries. Within 8 h blood flow in glomerular capillaries also did shutdown, as noted by their lack of red dextran and/or flowing erythrocytes (Figure 1B). At these later time points (7–8 h) faint red staining, originating from the 500 kDa dextran vasculature marker, was observed within the S1 segment of the proximal tubule indicating a breakdown in the glomerular capillary filtration barrier (Figure 1B, 8 h). These data indicate that UPEC express the appropriate attachment organelles to mediate colonization of the glomerulus despite the shear stress of filtrate flow. This implies that the epithelial composition is not the defining factor for lack of glomerular colonization during pyelonephritis.

### UPEC express both P and Type 1 fimbriae during *in vivo* kidney infection

**Figure 2:**
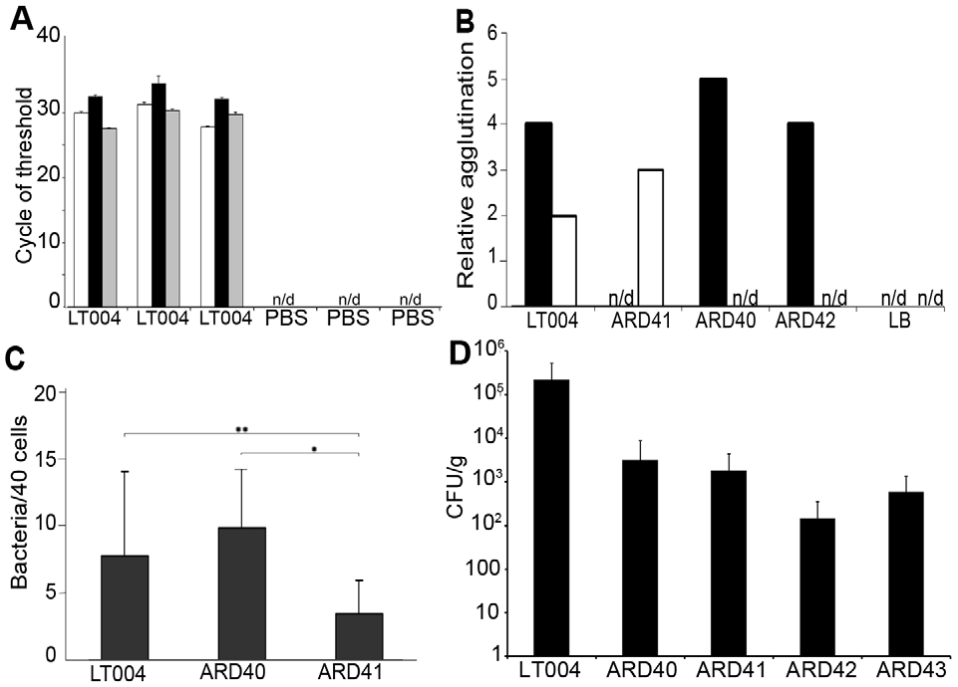
Expression, binding and infection characteristics of P and Type 1 isogenic strains. (**A**) qRT-PCR analysis of micro-dissected tissues 8 h after infusion with LT004 or PBS. Bars show cycle of threshold for detection of *gfp^+^* (white), *papA_2* (black) and *fimA* (grey) transcript. n/d  =  not detectable. Each group is from an individual animal (**B**) Relative agglutination of human O type red blood cells, indicating PapG mediated agglutination (black) and yeast cell agglutination in the absence of mannose, indicating FimH mediated agglutination (white) for indicated strains using LB medium as control. (**C**) Bacterial adhesion (counted per 40 cells) to A498 human renal epithelial cells. * P = 0.001, ** P = 0.045 Error bars in (a, c)  =  standard deviation. (**D**) Number of bacteria in the kidney, shown by CFU/g tissue, following a 4-day ascending infection.

Genetic analysis of the expression pattern of Type 1 and P fimbriae in carefully controlled *in vitro* experiments has shown that an individual *E. coli* bacterium express only one fimbriae type at a time due to co-regulation of the fimbriae operons [28], [29], [30]. To analyze the gene expression patterns of Type 1 and/or P fimbriae by UPEC colonizing the renal tubule, the spatial-temporally controlled micro-infusion model was used. Tissue infused with LT004 8 h previously was carefully excised and bacterial mRNA was isolated. qRT-PCR analysis revealed substantial expression of both major fimbriae structural proteins PapA_2 and FimA (Figure 2A). This suggested a bacterial population with heterogeneous expression of adhesion organelles at this early stage of infection.

### P fimbriae expression enhances early attachment and colonization to the tubular epithelium

**Figure 3:**
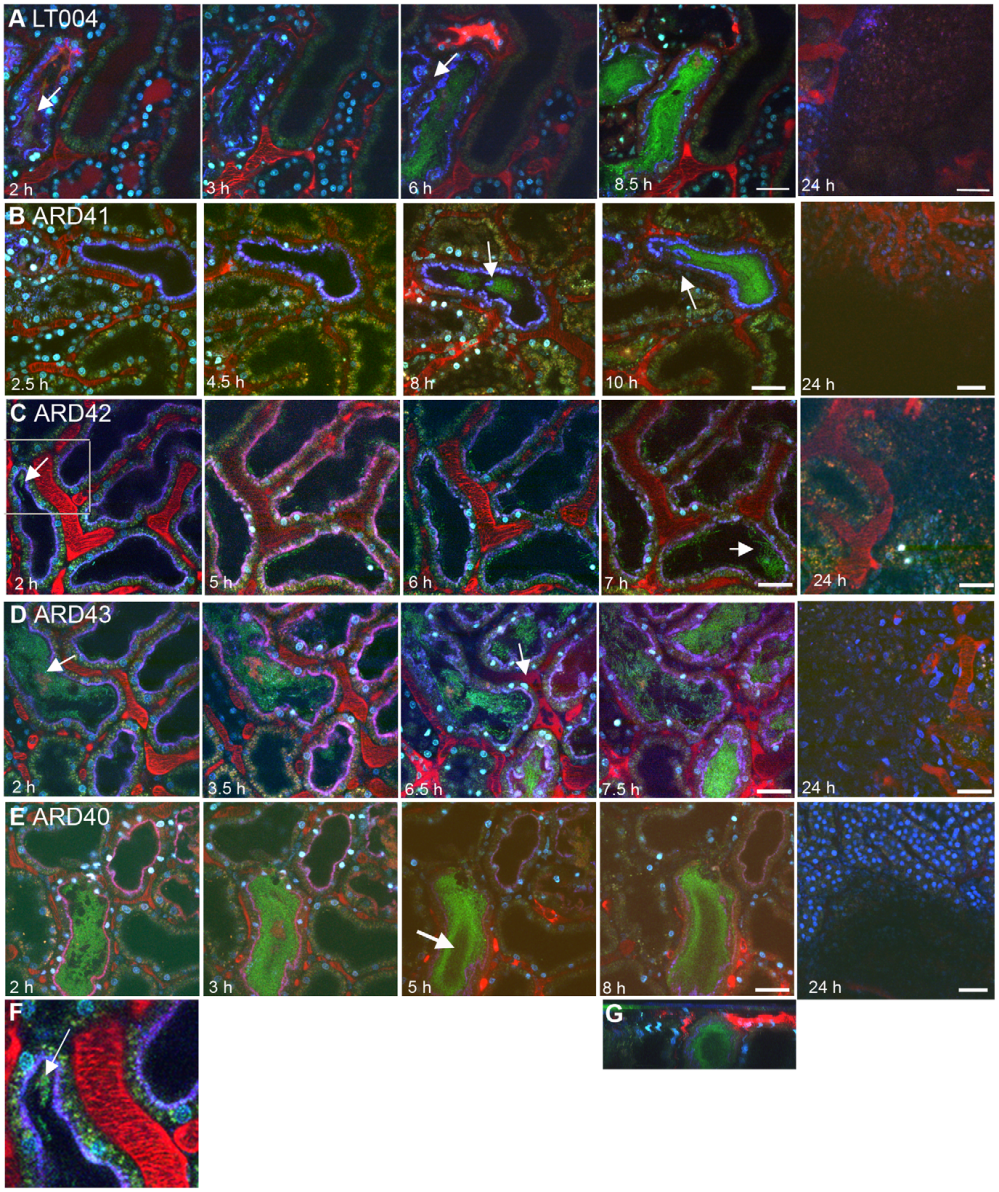
Progression of infection in live animals. Multiphoton imaging of renal tissue after infusion of indicated strains (green). Injected proximal tubules are outlined by a co-injected 10 kDa cascade-blue dextran (blue); blood plasma is labeled with 500 kDa rhodamine dextran (red) and cell nuclei are stained with Hoechst 33342 (cyan). (**A**) Wild type LT004 bacteria (green, arrow) (n = 12) can be seen colonizing the infected tubule (blue outline) within 2 h. As bacteria multiplied, shutdown of the peritubular capillaries was observed by a loss of the red plasma marker within surrounding capillaries (arrow, 6 h). (**B**) The UPEC strain ARD41, which lacks PapG mediated attachment, showed compromised colonization kinetics with few bacteria visible before 8 h (arrow). At 10 h, bacteria colonized the tubule lumen and signs of vascular dysfunction appeared (arrow) (n = 12). (**C**) The *E. coli* K-12 strain ARD42 (n = 5) showed a delayed colonization, with few bacteria colonizing the tubule at 2 h (arrow and insert, which is shown as Figure 3F). Colonization at 7 h was less as compared to LT004 (arrow). (**D**) Expression of P fimbriae in strain ARD43 restores colonization kinetics. Arrow at 2 h identifies bacterial colonization. At 6.5 h the beginning of vascular dysfunction can be seen by the lack of black shadows typical of flowing erythrocytes (arrow) (n = 4). (**E**) Lack of FimH mediated attachment in UPEC strain ARD40 shows that bacteria struggle to maintain themselves in the center of the tubule lumen, with less bacteria in the center of the tubule (arrow 5 h) (n = 7) (**F**) 50 µm wide inset from Figure 3C showing bacterial colonization of the tubule. (**G**) shows x-z projection of panel 3E, ARD40 8 h, demonstrating the bacterial ‘tube’ structure. Images from 24 h show bacterial clearance and edema formation in all strains. Scale bars: 7–10 h  = 30 µm, 24 h  = 50 µm. All figures presented are representative images, displaying the characteristic colonization patterns for each experimental set.

We then analyzed the role of the well-known UPEC adhesion factor P fimbriae during *in vivo* colonization. To do this we used strain ARD41, a GFP^+^ expressing derivative of CFT073 containing defined mutations in both copies of *papG* (Table 1). ARD41 therefore lacked P fimbriae mediated attachment, but could still express functional Type 1 fimbriae. Phenotypic analysis of this strain demonstrated the expected erythrocyte and yeast agglutination pattern (Figure 2B), and a significant reduction of bacterial binding to A498 renal epithelial cells *in vitro* (Figure 2C). In an ascending model ARD41 was able to ascend and infect the kidneys, though a reduced number of bacteria, shown as CFU/g tissue, was demonstrated as compared to the UPEC strain LT004 (CFT073 GFP^+^) (Figure 2D). Real-time analysis of the renal infection process was performed using multiphoton microscopy. These dynamic *in vivo* imaging experiments revealed that this strain, lacking P fimbriae, colonized less efficiently as it only established infection in approximately 33% of infusions, as compared to an approximate 95% success rate for LT004. In successful ARD41 infections, the absence of P fimbriae delayed colonization of the tubule to 7–8 h post infusion (Figure 3B) in comparison to the wt strain, which showed colonization within 2 h of infection (Figure 3A). We have reported previously the shutdown of local peritubular capillaries as a response to tubular infection [20]. In ARD41 infections the vascular shutdown response, relative to bacterial load, was slower than that seen for LT004, but yet visible (Figure 3B 10 h arrow). The spatial-temporal precision of this infection model allows for the exposed kidney to be returned to the peritoneal cavity and re-analyzed on subsequent days. MPM-analysis of the infection site 24 h post-infusion showed that bacteria had been cleared, leaving cortical edema and extensive tissue destruction (Figure 3B 24 h), the same outcome as seen for LT004 infections (Figure 3A 24 h).

**Table 1:** Bacterial strains and oligonucleotides used in this study.

Strain	Genotype	GFP^+^	FimH	PapG	LPS	Reference
LT004	CFT073 *cobS::*Φ(*P_LtetO-1_-gfp^+^*), *cm^r^*	X	x	x	Smooth	[19]
UPEC76	CFT073 Δ *papDEFG* (*pheV)*Δ *papEFG* (*pheU)*	-	x	-	Smooth	[10]
W3110	*E. coli* K-12 Lab strain	-	x	-	Rough	[55]
ARD40	LT004 *fimH*::*km^r^*	X	-	x	Smooth	This work
ARD41	UPEC76 *cobS::*Φ(*P_LtetO-1_-gfp^+^*), *cm^r^*	X	x	-	Smooth	This work
ARD42	W3110 *cobS::*Φ(*P_LtetO-1_-gfp^+^*), *cm^r^*	X	x	-	Rough	This work
ARD43	ARD42 pKTH3020	X	x	x	Rough	This work
MC1	*Salmonella enterica* serovar *typhimurium*					[56]
**Plasmid**						
pKTH3020	*pap* operon from KS71			x		[*57*]
pKD4	Km resistance cassette source					[54]
pKM001	P*_LtetO-1_*-GFP^+^ source plasmid					[19]
**Oligos**						
FimH KO F^a^	ATTACCCAGAAACCATTACAGACTATGTCACACTGCAACG*TGTGTAGGCTGGAGCTGCTTC*					
FimH KO R^a^	CATCATTATTGGCGTAAATATTCCACACAAACTGGAAATC*CATATGAATATCCTCCTTAG*					
FimA F	GCTCTGTCCCTCAGTTCCAC					
FimA R	TCAACAGAGCCTGCATCAAC					
PapA F	TGAAATTCGCAACTGCTGAG					
PapA R	AACGGGTGAAATTTGATGGA					
GFP+ F	ATCCGTTCAACTAGCAGACC					
GFP+ R	GTTACAAACTCAAGAAGGACCA					

a 40 bp homology to *fimH*, followed by 20 bp homology to pKD4 (italics).

The role of P fimbriae in early stage epithelial colonization was further strengthened by experiments using an isogenic set of *E. coli* K-12 strains that did and did not express P fimbriae. By complementing a GFP^+^ expressing *E. coli* strain W3110 (ARD42) with a plasmid encoding the *pap* operon, we obtained isogenic strains that did (ARD43) and did not (ARD42) express P fimbriae (Table 1). Their respective P fimbriae phenotype was verified in erythrocyte agglutination assays and Type 1 fimbriae expression was confirmed with a mannose independent yeast agglutination assay (Figure 2B). These strains also lacked many other known virulence factors such as the α- hemolysin toxin (Hly). To test the relevance of these K-12 strains in urinary tract infection, 10^8^ CFU was inoculated into bladders in an ascending model of UTI. Both strains were found to ascend to and infect the kidney, though lower bacterial numbers were observed in the tissue as compared to UPEC strains (Figure 2D).

In the live imaging model ARD42, which lack P fimbriae, showed delayed tubular colonization kinetics as it was only able to initiate colonization 6–7 h after infusion (Figure 3C,3F). The colonization kinetics were rescued in strain ARD43, which over-expresses plasmid encoded P fimbriae. ARD43 showed greater initial colonization than ARD42 (Figure 3D) and populated the tubules with similar kinetics to LT004 (Figure 3A). The vascular shutdown following infection with ARD43 was however delayed in comparison to infection with UPEC strain LT004 (Figure 3D). This difference may stem from a lack of expression of other virulence factors such as Hly, known to effect vascular shut-down kinetics [19]. At 24 h post-infusion bacterial clearance, edema formation and tissue damage was observed irrespective of P fimbriae expression, implying that the delayed colonization kinetics of ARD42, lacking P fimbriae, is overcome within 24 h (Figure 3C, D 24 h). These results corroborate the findings for ARD41 that P fimbriae enhance the early stage of tubule colonization. They also indicated that while *E. coli* K-12 strain does not elicit the same rapid host response as UPEC, it can cause inflammation and edema over 24 h.

Dynamic imaging of the infection revealed a striking feature only observed in the two strains lacking the PapG tip adhesin. Both ARD41 and ARD42 were observed being ‘flushed’ through the tubule by the filtrate flow. Video S1 shows a large bacterial mass moving in a tubule, indicating that bacteria lacking P fimbriae appear to be more susceptible to filtrate flow. Figure S1 shows a tracing of this video showing the approximately 70 µm path the bacterial mass moves during the 70 s duration of the video. In our studies, we observed this event 3–4 times in multiple animals during independent infections. Due to the speed and unpredictability of this event the possibilities of capturing it at a certain time point is limited and therefore these numbers are probably under-representative of the occurrence of this event. Together these data suggest that expression of P fimbriae provides a fitness advantage *in vivo*, aiding bacterium in withstanding the filtrate flow and enhancing colonization during the first hours of infection.

### FimH mediates inter-bacterial binding and bacterial colonization of the center of the renal tubule

A similar analysis was performed to investigate the role for Type 1 fimbriae in early colonization. An insertion deletion was introduced into the *fimH* of the GFP^+^-expressing derivative of CFT073, strain LT004 (Table 1). Successful inactivation, demonstrated in erythrocyte and yeast agglutination experiments (Figure 2B) suggests that the resulting strain ARD40 lack the ability to bind via Type 1 mediated attachment. In motility assays, LT004 showed an average swimming diameter of 26±1.5 mm and ARD40 22±1.7 mm (*p* = 0.116), indicating that the absence of the FimH tip adhesin did not influence bacterial motility. ARD40 was also able to infect the kidneys in the ascending model, but again showed lower numbers of bacteria in the tissue than LT004 (Figure 2D). Dynamic *in vivo* imaging showed that ARD40 colonized the tubule at a level comparable to LT004, and the pathophysiology at 24 h was similar to that induced by the other strains (Figure 3E). Type 1-negative bacteria did, however, display a unique feature. While bacteria efficiently colonized along the tubular epithelium, the bacterial density in the centre of the lumen was dramatically reduced (Figure 3E, 5 h). Hollow “bacterial tubes” appeared to form (Figure 3G), suggesting that in areas where bacteria have no epithelium on which to adhere they have difficulty maintaining themselves.

**Figure 4:**
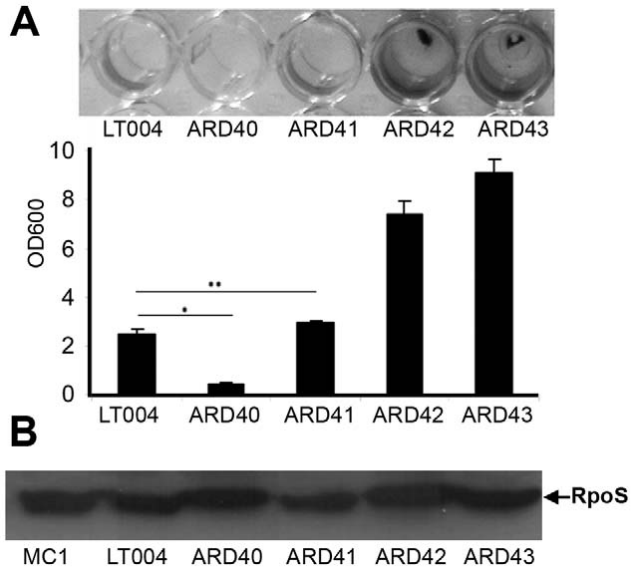
UPEC biofilm formation. (**A**) Crystal violet assay of biofilm formation from indicated strains. Visualization (top) and quantification (bottom) of biofilm at OD_600_. Error bars  =  standard deviation. * P = 0.001 ** P = 0.032 (**B**) Western blot of RpoS protein in indicated strains using MC1 as positive control.

In perfused environments, microbial communities are established via a process known as “self-immobilization” [31], [32]. Sessile biofilms are formed as bacteria embed themselves in an endogenously formed matrix. This compact community, consisting of organisms' adherent to each other and/or a surface, provides extraordinary resistance to hydrodynamic flow shear forces. The FimH adhesin has been shown to be instrumental in biofilm formation by *E. coli* K-12 under both static and hydrodynamic growth condition *in vitro* [33], [34]. This suggested that the Type 1 fimbriae may confer on UPEC the ability to form biofilm that opposes bacterial clearance from the central part of the tubule lumen. The biofilm forming ability of strains included in this study was visualized and quantitated *in vitro* using polystyrene microtiter plate assays (Figure 4A, B). The wt UPEC strain LT004 as well as the *papG* mutant strain ARD41 both formed low, yet notable amounts of biofilm. This was in contrast to the *fimH* mutant strain ARD40, whose biofilm-forming capacity was significantly reduced. As expected, the *E. coli* K-12 strain ARD42 (expressing Type 1 fimbriae) showed robust biofilm formation, which was unaffected by P fimbriae complementation (ARD43). *Salmonella enterica* serovar *typhimurium*, a known biofilm forming strain, was included as a positive control. A Western blot revealed that all strains expressed RpoS, the master regulator of general stress responses which has previously been shown to effect biofilm formation [35] (Figure 4B). These results suggest that FimH does play a role in UPEC biofilm formation and may imply that the lack of ARD40 colonization of the tubule center is related to this strain's inability to mediate inter-bacterial binding and biofilm formation in this perfused micro-environment.

### Infection contributes to nephron obstruction

**Figure 5:**
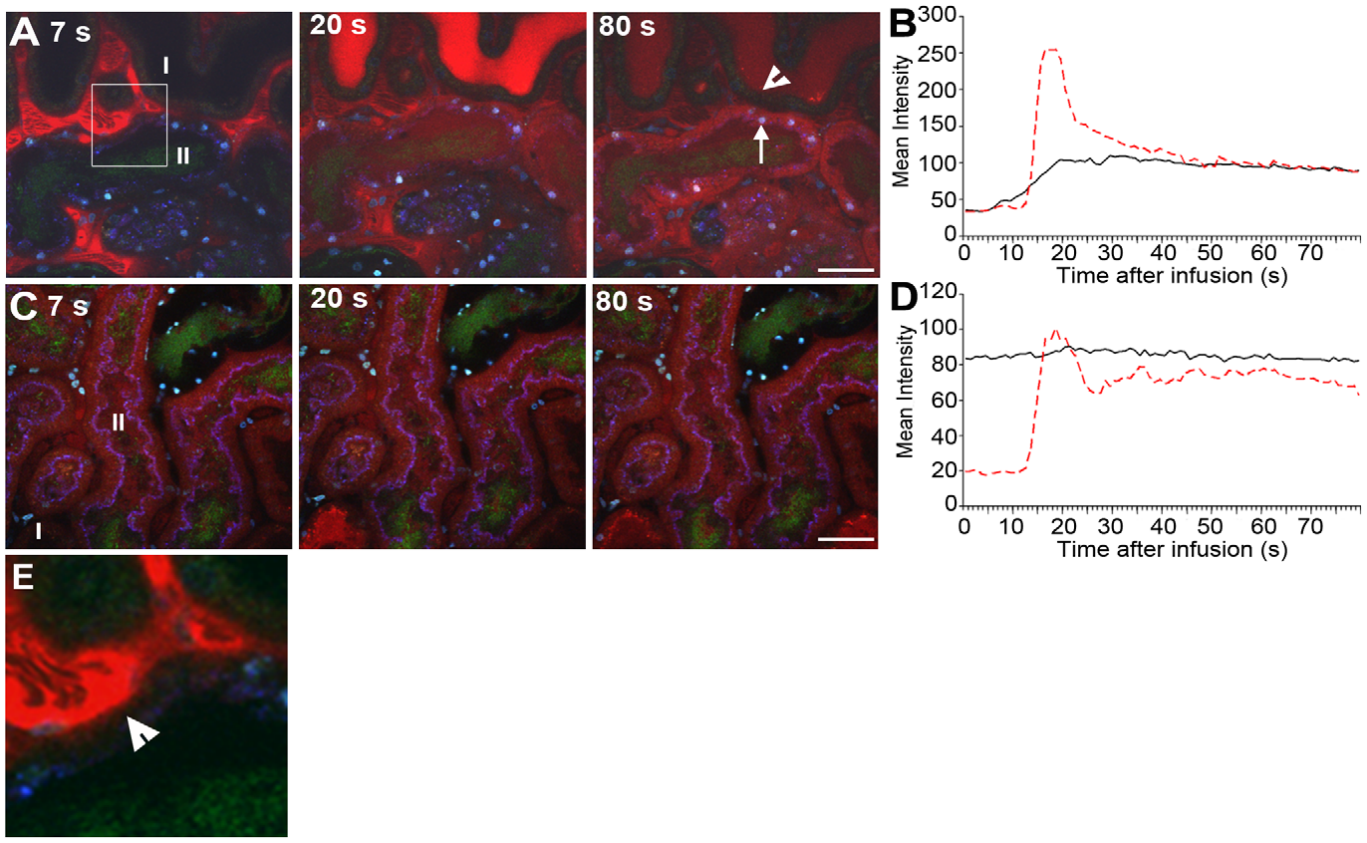
Infection affects renal filtration. Glomerular filtration in non-infected (I) and infected (II) tubules 4 h **(A,B)** and 8 h **(C, D)** after LT004 infusion is visualized 7, 20, and 80 s after iv bolus infusion of 10 kDa dextran (red). These data sets are representatives from a single animal, the experiments were performed on at least 3 separate occasions. The dynamic aspect of renal filtration and clearance can be seen in Videos S2 and S3, from which these frames originate. (**A**) Efficient filtration of the non-infected nephron (I) is visualized by the appearance of the bright red dextran in the tubule lumen (20 s), followed by a drop in intensity (80 s) indicating clearance. A less dramatic intensity change is seen in the infected nephron, indicating less filtration. Epithelial dextran uptake (arrow, 80 s) indicates epithelial dysfunction in infected tubule. Healthy epithelia in non-infected tubule exclude the dextran (arrowhead, 80 s). (**B**) Quantification of the mean intensity of dextran in tubule lumens over 80 s. Dotted red line corresponds to luminal intensity in the non-infected tubule (I), black line corresponds to the infected tubule (II). (**C–D**) The absence of filtrated dextran in the infected tubule (II) demonstrates compromised filtration at 8 h. Tissue shows accumulation of some dextran from previous bolus infusions. Scale bars  = 30 µm. (**E**) Enlarged 50 µm inset from 5A 7 s showing dextran leaking into epithelial cells (arrowhead) from the basal side of the cells.

One may envisage that formation of dense bacterial communities within the tubular lumen would influence renal filtrate flow. The effect of infection on filtrate flow was analyzed by systemic injections of small molecular weight red fluorescent dextran. After filtration by the glomerulus, dextran appears within the tubule lumen where it can be used to visualize filtrate flow [36]. Four hours after micro-infusion of LT004, a bolus of dextran was delivered intravenously until fluorescence in Bowman's space reached saturation. Within seconds of infusion, red dextran appeared within peritubular capillaries (Figure 5A, Video S2). A representative animal is shown in Figure 5. In non-infected nephrons, dextran was rapidly filtered and passed swiftly through the tubule lumens. This is visualized in Figure 5A (I), with quantification of this tubule's fluorescence shown in Figure 5B. Analysis of the infected nephron within the same field-of-view revealed lowered peak intensity, indicating a degree of obstruction and reduced glomerular filtration (Figure 5A II; Figure 5B). Repeating the experiment 8 h post-infusion, when the entire tubule lumen of the infected nephron was colonized by bacteria, showed that filtrate flow was completely obstructed and peritubular capillary blood flow was shut-down (Figure 5C II; Figure 5D, Video S3). In contrast, non-infected neighboring nephrons still displayed filtration (Figure 5C I; Figure 5D, Video S3). Similar experiments were performed using the isogenic mutant strains (data not shown). Obstruction was observed in these infections, but variability between animals prevented satisfactory statistical quantification and we were therefore unable to note any significant variation in the early phases of obstruction.

In addition to obstruction, local vascular leakage occurred as the bacterial infection progressed. Loss of epithelial membrane barrier function could be identified 4 h post-infusion, when dextran was found leaking into the epithelial cells of the infected tubule (Figure 5A, 80 s, arrow and Video S2). Careful inspection of data from the dynamic imaging (Video S2 and Figure 5E, which is an inset from Figure 5A 7 s) shows that leakage appears to start from the basolateral side of the epithelium. This is in contrast to the neighboring, non-infected nephrons, which maintain their epithelial barrier function at this stage of infection (Figure 5A, 80 s, arrowhead). At 8 h, non-infected neighboring tubules also show some signs of epithelial barrier function breakdown, likely linked to an ischemic injury [20] (Figure 5C, Video S2).

## Discussion

The ability to monitor real-time progression of bacterial infections in a living animal is providing a new foundation for microbial pathogenesis research. As the experiment is performed within the live organ, the roles of bacterial virulence factors can be studied *in vivo* in the presence of all physiological parameters. While recently reported real-time live animal infection models hold numerous advantages [37], [38], the experimental models can be very complex. One concern can be the route of delivery of the infecting agent. Experimental control over the spatial and temporal aspects of the infection is of utmost importance to allow for time-resolved studies. In the MPM model presented here bacteria are microinfused directly into the kidney nephron. This evidently differs from the natural ascending route of infection but is essential to allow for imaging of the infectious time course starting from the first host-pathogen interaction. In this, as well as our previous studies [14], [19], [20] we have shown that the majority of infused bacteria are immediately flushed from the tubule and the visualized infection stems from the very few bacteria that initially bind and adapt to the tubule microenvironment.

The findings presented here suggest that UPEC's attachment organelles, P and Type 1 fimbriae, act synergistically to facilitate bacterial colonization in the face of challenges such as renal filtrate flow (see an animated summary in Video S4). In ascending UTI models we and others [10] have shown that PapG, the tip adhesin of P fimbriae, is not essential for renal infection with CFT073.The temporal control of the MPM approach used here revealed that expression of this adhesin enhances the colonization kinetics during the very early, first hours of infection. In the living animal model P fimbriae appear to promote epithelial attachment and resistance to filtrate flow, facilitating early bacterial multiplication prior to the onset of ischemia and infiltration of immune cells [20]. These findings suggest P fimbriae may function as a ‘fitness factor’ in much the same way as siderophores. Iron sequestering sideophores have been annotated as fitness factors because while their expression is not essential to bacterial virulence, it is shown to be advantageous [39], [40]. Considering P fimbriae as a fitness factor *in vivo* may rationalize it's over-representation in clinical UPEC isolates as well as explaining its presence in many, but not all, pyelonephritis isolates [8].

Contrary to the widespread view that Type 1 fimbriae plays its primary role in cystitis, our data indicates it also plays a key role in kidney infections. Our data are the first to indicate that Type 1 fimbriae facilitate inter-bacterial adhesion and biofilm formation, allowing bacteria to maintain themselves within the center of the tubule lumen. A lack of FimH mediated inter-bacterial binding reduced the bacteria's ability to colonize the center of the tubule, where they have no epithelium on which to attach. This hypothesis is reinforced by previous findings which demonstrate the importance of the FimH adhesin in *in vitro* biofilm formation under shear stress [34]. In strain ARD41, which expresses Type 1 fimbriae but lacks PapG, bacterial colonization was at later stages of infection visualized in the center of the tubule lumen, away from the epithelium (See Figure 3B, 8 h). This pattern shows similarities to anaerobic upflow systems where microbes form dense spherical biofilm aggregates within the liquid by a process known as self-immobilization [31]. These aggregates consist solely of microorganisms, which themselves produce the matrix in which they are embedded [31], [32]. Self-immobilization was proposed by Sonnenburg and co-workers as the mechanism allowing bacterial communities to establish themselves independently of epithelial attachment in perfused environments such as the lumen of the intestine [32]. It may be that similar mechanisms operate within the tubule lumen, with FimH facilitating inter-bacterial adhesion by binding to mannose present within the LPS of neighboring bacteria [41]. This inter-bacterial binding or biofilm formation would facilitate luminal colonization, while a planktonic form of bacteria would be rapidly removed by the filtrate flow. UPEC strains have previously been shown to form biofilms on catheters as well as on and in the bladder epithelium [42], [43], whereas our data suggests a new role of UPEC biofilm in renal infection. The relevance of this biofilm formation in human pyelonephritis remains to be investigated. The synergistic action of P and Type 1 fimbriae we observe supports a previous concept that bacteria generate a heterogeneous fimbriae population to cope with the unpredictability of their environmental niche [28]. Our data further implies that these niches may not only be as large as between the kidney and bladder but as small as between the centre and periphery of a single tubule lumen.

The role of shear stress on bacterial adhesion and colonization is becoming increasingly appreciated. Recently a relationship between the *E. coli* FimH protein and shear stress was reported [44], [45], [46], [47], showing that the binding strength of FimH to mannosylated surfaces is enhanced by shear. This is mediated via a force-enhanced allosteric catch-bond, which functions via a finger-trap like β sheet twisting mechanism [44]. At a shear of 0.02 dynes/cm^2^ the binding strength of FimH was considered weak whilst as the shear increased to 0.8 dynes/cm^2^ this binding become stronger [47]. Shear stress in the renal proximal tubules has been reported to be 0.17 dynes/cm^2^, though the fluctuating nature of fluid flow as well as tubular absorption and secretion may imply a degree of variability in this rate [48]. The apparent low level of shear stress present in the renal tubule may mean that the catch-bond mechanism of FimH plays a less significant role in this niche than in the bladder where shear stress may be higher upon voiding. A low shear, coupled with the lack of uroplakin monomannose residues on renal epithelial cells, may also help explain the low level of FimH mediated epithelial binding in the early stages of infection. The same laboratory has also shown that UPEC positively select for a FimH variant that maintains attachment following a drop in shear, as compared to fecal or vaginal *E. coli* isolates [49]. This variation in the signal peptide of FimH, which results in expression of less, though longer fimbriae, may be more relevant under the fluctuating conditions UPEC faces *in vivo*. Conversely PapG has been shown to mediate binding under both shear force as well as in static conditions [50]. While the reported shear stress value for the renal tubule is low in regards to the *in vitro* data of Thomas *et al* [46], [47], the supplementary videos presented in this study, (Videos S1 and S3) do demonstrate that the niche is far from static in the early phases of infection. Further investigation is needed to draw any definitive conclusions between physiological shear in the renal tubule and the catch-bond mechanisms of *E. coli* adhesions. Interestingly FimH is present in all virotypes of *E. coli* [51], and a role for FimH in inter-bacterial binding may explain a general function for the Type 1 fimbriae in diverse perfused environmental niches.

Filtrate flow is an oft overlooked, yet crucial factor of the *in vivo* environment. Renal obstruction injury in itself is known to cause hemodynamic changes, epithelial damage, loss of cell membrane integrity and the expression of a number of inflammatory mediators [52], [53]. We previously showed that bacteria located in the tubule activates the clotting cascade in peritubular capillaries leading to local reduction of blood flow, PO_2_ and subsequent ischemic damage [20]. The present study adds obstruction to the list of contributing factors resulting in the full pathophysiology of renal infection. The complete stoppage of filtrate flow through the nephron occurring only hours after bacterial exposure is likely to result from a combination of physical obstruction by the bacteria, reduction in blood flow to the area, and the death and exfoliation of proximal tubule cells related to the ischemia. If the obstruction is related to the ischemic response it may assist in isolating the infection and preventing further bacteria spread. As with other host defense mechanisms, such as neutrophil bursts and the ischemic response, a certain level of collateral damage to the tissue is inevitable. These findings may also have important clinical consequences such as difficulties in delivery of systemically injected antibiotics to the infected nephron. Further studies are needed to define the précis signaling events occurring following infection. Collectively, our work presented here illustrates that dynamic imaging within a live setting has great potential to define new physiologically/clinically relevant outcomes of the complex microbe-host interplay.

## Methods

### Ethics statement

All studies were performed in accordance with the National Institutes of Health's *Guide for the Care and Use of Laboratory Animals* and have been approved by the Institutional Animal Care and Use Committee at Indiana University School of Medicine Indianapolis, Indiana, USA (Study number 2453 Amendments 4, 5 and 16), Uppsala Djurfösöksetiska Nämnd, Uppsala, Sweden (Permit number: C14/6) or Stockholm's Norra Djurförsöksetiska Nämnd, Stockholm, Sweden (Permit numbers: N190/05, N347/09, N402/07).

### Bacterial strains and plasmids

Bacterial strains used in this study are listed in Table 1. Strains ARD41 and ARD42 were constructed by inserting the *gfp* ^+^ gene into UPEC76 and W3110 as previously described [19]. Briefly, the one-step allelic recombination method was used to achieve site-specific integration of *gfp^+^*, under the control of a constitutively active tetracycline promoter P_LtetO-1,_ into the *cobS* gene [54]. To generate strain ARD43, plasmid pKTH3020, carrying the *pap* operon, was inserted into strain ARD42 by electroporation. ARD40 was created by inserting the kanamycin resistance cassette from pKD4 into the *fimH* gene of LT004 deleting 321 bp between nt 5143780- 5144101, using the one step allelic knockout method [54]. Oligonucleotide sequences are listed in Table 1. All insertions were confirmed by PCR and sequencing (ABI3100, Applied Biosystems). For cloning purposes, bacteria were cultivated in aerated Luria-Bertani broth at 37°C in the presence of ampicillin (Amp, 100 µg/ml), chloramphenicol (Cm, 20 µg/ml) and kanamycin (Km, 50 µg/ml) as required. No alterations in growth rates, capsule morphology or expression of α-hemolysin were observed (data not shown). To prepare bacteria for microinfusion experiments, bacteria from aerated over-night cultures were re-inoculated (1∶100) into fresh LB with antibiotics, cultivated under shaking conditions to OD_600_  = 0.6, then washed and concentrated to 10^9^ CFU/ml in PBS. Bacteria were maintained on ice (maximum 2 h) until microinfusion.

### mRNA analysis

The renal infection site was dissected using a 5 mm biopsy punch, medulla tissue was removed, and total RNA extraction was performed on the resulting ∼30 mg tissue using Trizol (Invitrogen, Sweden). Experimental triplicates were performed on three separate preparations for both LT004 infected and PBS samples. cDNA was transcribed from 1 µg RNA using the SuperScript III First Strand Synthesis Supermix kit (Invitrogen, Sweden). qRT-PCR was performed using a 7500 Real Time PCR System (Applied Biosystems, Sweden) and the Power SYBR Green PCR Mastermix (Applied Biosystems, Sweden). In all experiments, *gfp* ^+^ was used as an endogenous reference gene. Primer sequences are listed in Table 1.

### Adhesion assays

The human kidney epithelial cell line A498 was grown on coverslips in 24-well cell culture plates in RPMI-1640 media with 10% FCS and 2 mM L-glutamate. Cells were infected with 10^5^ CFU/ml LT004, ARD40, or ARD41 for 30 min at 37°C, 5% CO_2_, 95% humidity. Cells were washed 2×5 min in PBS and fixed in 4% paraformaldehyde before microscopic analysis. Image J (U. S. National Institutes of Health, MD, USA, http://rsb.info.nih.gov/ij/) was used to evaluate bacterial attachment per 40 cells. Data is pooled from a minimum of 11 tests from 2 independent experiments. Statistical significance was tested using the Student's T-test.

### Agglutination assays

To detect PapG mediated agglutination, bacteria were grown overnight on a LB agar plate at 37°C. Agglutination was performed with human RBCs (O type) (8% vol/vol in PBS). To detect FimH mediated agglutination bacteria were grown overnight in a static LB culture at 37°C. Bacteria were added to a yeast suspension (5% vol/vol PBS) in the absence of mannose.

### Ascending infections

Bladders of isofluran-anesthetized female Sprauge-Dawly rats (200 g) (B and K Universal AB) were catheterized and 10^8^ CFU of the respective bacterial strains in 1 ml PBS or control PBS were slowly infused. All strains were introduced into 5 separate animals (n = 5). Four-days post infection animals were sacrificed and kidneys removed. Kidneys were homogenized and CFU counts were obtained by plating on selective LB agar plates containing appropriate antibiotics.

### Microinfusion procedures

Microinfusion infection was carried out as previously described [19]. Bacteria (10^9^ CFU/ml in PBS) were mixed with 1 mg/ml Fast Green FCF (Fisher, Fair Lawn, NJ, USA) and 0.2 mg/ml Cascade blue-conjugated 10 kDa dextran. Bacteria or PBS control suspensions were aspirated into sharpened micropipettes filled with heavy mineral oil. Male Sprague-Dawley (269±30 g) or Munich-Wistar (240±80 g) rats were anesthetized by intra-peritoneal injection of 40–50 mg/kg sodium pentobarbital or 130–150 mg/kg thiobutabarbital (Inactin) (Sigma, St. Louis, MO). Munich-Wistar rats were used to allow for Bowman's capsule injections, and glomerular imaging, due to their surface glomeruli. Surgical procedures performed included a tracheotomy and cannulation of femoral artery, femoral vein and the jugular vein. The left kidney was exposed via a subcostal flank incision, and gently exteriorized. The kidney was supported by a shaped cup and using a Leitz micromanipulator and mercury leveling bulb under stereoscopic microscope observation (96×), the bacterial suspension was infused over a period of 10 min. To allow localization of injection sites Sudan black-stained castor oil was injected into nearby tubules. Injections were performed into either the proximal tubules (LT004 n = 15, ARD40 n = 7, ARD41 n = 12, ARD42 n = 5, ARD43 n = 4, and PBS n = 20) or Bowman's space (LT004 n = 5 and PBS n = 3). Bacteria were infused at an average rate of 43 nl/min corresponding to delivery of 3–6×10^5^ CFU per injection.

### Multiphoton microscopy

All multiphoton imaging was performed using the set-up previously optimized and described [19]. Images were collected using a Bio-Rad MRC 1024 confocal/2-photon system (Bio-Rad, Hercules, CA) attached to a Nikon Diaphot inverted microscope (Fryer Co, Huntley, IL) with either a Nikon ×60 1.2-NA water-immersion or a 20x objective. Fluorescence excitation was provided by a Tsunami Lite titanium-sapphire laser (Spectraphysics, Mountain View, CA). Image stacks were collected in 1 µm optical steps into the tissue at a depth of approximately 30-100 µm using an excitation wavelength of 810 nm and neutral density filters set to 25–40%. Fluorescent probes were injected as a single bolus via a jugular vein access line. Tetramethylrhodamine-conjugated 500 kDa dextran (∼2.5 mg/400 µl 0.9% saline, Molecular Probes, Eugene, OR) was used to visualize blood flow and Hoechst 33342 (∼600 µg/0.4 ml of 0.9% saline, Molecular Probes, Eugene, OR) to stain cell nuclei. To image filtrate flow 10,000 kDa texas-red dextran was infused via the jugular vein access line until the concentration in Bowman's space reached saturation.

Anesthetized rats were placed on the microscope stage with the exposed kidney positioned in a 50 mm-diameter coverslip-bottomed cell culture dish (Warner Instruments, CT, USA) containing isotonic saline. Body temperature was monitored rectally and maintained using a heating pad covering the rat. Arterial blood pressure was regularly monitored and the rat continuously infused, via a femoral venous line, with normal saline (0.9%, 1.5 ml/h) using a syringe pump (Sage Instruments, Freedom CA). During the experiments control regions of either PBS infusion or naïve cortex were routinely checked to verify both fluorescent signal and healthy renal function.

### Image processing

Images and data-volumes were processed using Metamorph Image Processing Software (Universal Imaging-Molecular Devices, PA, USA) and Image J (U. S. National Institutes of Health, MD, USA, http://rsb.info.nih.gov/ij/). Final figures were prepared with Adobe Photoshop (Adobe, CA, USA). All figures presented are representative images from each experimental set (n numbers listed above).

### Biofilm analysis

The bacterial strains were diluted 1∶10 from an LB overnight culture (37°C) to LB medium without NaCl. 0.2 ml was added into the wells of a 96-well microtitre plate, which was incubated at 28°C for 24 h. Following incubation medium containing the planktonic bacteria was decanted and wells were washed three times with PBS. Bacteria attached to the walls of the wells were stained by adding 250 µl/well of crystal violet and incubated 10 min (room temperature) before decanting and drying. Biofilm was imaged using a digital camera. Quantification was performed by dissolving the attached bacteria with 70% ethanol and measuring the optical density at 600 nm. All samples were analyzed in triplicate from three independent experiments, using Student's t-test.

Bacteria were grown on LB agar for Western blot analysis. Bacteria (5 mg wet weight) were harvested, re-suspended in sample buffer (0.5 M Tris-HCl, pH 6.8, 87% glycerol, 4% SDS, 0.2% bromphenol blue) and heated at 95°C, 10 min. To equalize the samples, protein content was adjusted using Coomassie blue staining (20% methanol, 10% acetic acid, 0.1% Coomassie brilliant blue G). Proteins were separated by sodium dodecyl sulphate-polyacrylamide gel electrophoresis (12% resolving gel with 4% stacking gel), and transferred to a polyvinylidene difluoride membrane (Hybond-P, Amersham Biosciences). Detection of RpoS was performed according to the manufacturer instruction using a primary anti-mouse monoclonal antibody (2G10, dilution 1∶10 000, NeoClone Biotechnology, Madison) and anti-mouse immunoglobulin G conjugated with horseradish peroxidase (1∶5000, DAKO A/S Denmark). Peroxidase activity on the Hyperfilm ECL (Amersham Biosciences) was recorded using LAS-1000 system (FUJIFILM).

### Motility assay

LT004 and ARD40 grown on LB agar plates overnight were re-suspended in water to OD_600_ = 0.6. 7 µl of the suspensions were inoculated into the swimming media (0.3% LB agar) and plates were incubated at 37°C for 16 h. The diameter of the swimming zone was then measured. Three experiments were performed with independent cultures in triplicate, and analyzed using Student's t-test.

### Accession numbers

CFT073 – Genebank AE014075, ref seq. NC-004431; E. coli K-12 W3110 AC_000091; fimH Gene ID1037233; pap operon Gene ID 1039518, CobS Protein ID AE016762_190.

## Supporting Information

Figure S1 Movement of ARD42 through a proximal tubule. (A) Image taken at the beginning of the video showing the position of the bacteria and a trace line showing the path they travel over the 70 s duration. (B) Image taken at the end of the video, 70 s later, showing bacterial position.
(0.75 MB TIF)

Video S1 Video showing movement of ARD42 (green) through a tubule (outlined blue) at 8 h post infection. Total capture time  = 60 sec (8.5fps).
(0.94 MB MOV)

Video S2 Video shows an infection with LT004 (green), 4 h post-infusion. Infected tubule in centre of frame is outlined with cascade blue (blue). 10 kDa dextran is injected intravenously at start of video. For details see Figure 5. Total capture time  = 80 sec (7.2 fps).
(1.10 MB MOV)

Video S3 Shows same LT004 infection as Video S2, 8 h post-infusion. 10 kDa dextran is injected intravenously at start of video. Total capture time  = 80 sec (7.2 fps).
(1.22 MB MOV)

Video S4 An animated summary of our hypothesis. This cartoon shows the bacterial infusion into a kidney tubule. A few of the infused bacteria attach to the tubule wall and begin colonization. During the early stages of infection the bacteria express P fimbriae to facilitate epithelial binding. As the bacteria colonize into the tubule centre Type 1 fimbriae expression becomes important in facilitation inter-bacterial binding. Synergy between these two fimbriae allows the bacteria to colonize the tubule and cause nephron obstruction.
(6.32 MB MOV)
