# Supplementary figures and images for: Uropathogenic Escherichia coli P and Type 1 Fimbriae Act in Synergy in a Living Host to Facilitate Renal Colonization Leading to Nephron Obstruction
Source: PLoS Pathog. 2011 Feb 24;7(2):e1001298. doi: 10.1371/journal.ppat.1001298 (PMC3044688; doi:10.1371/journal.ppat.1001298)

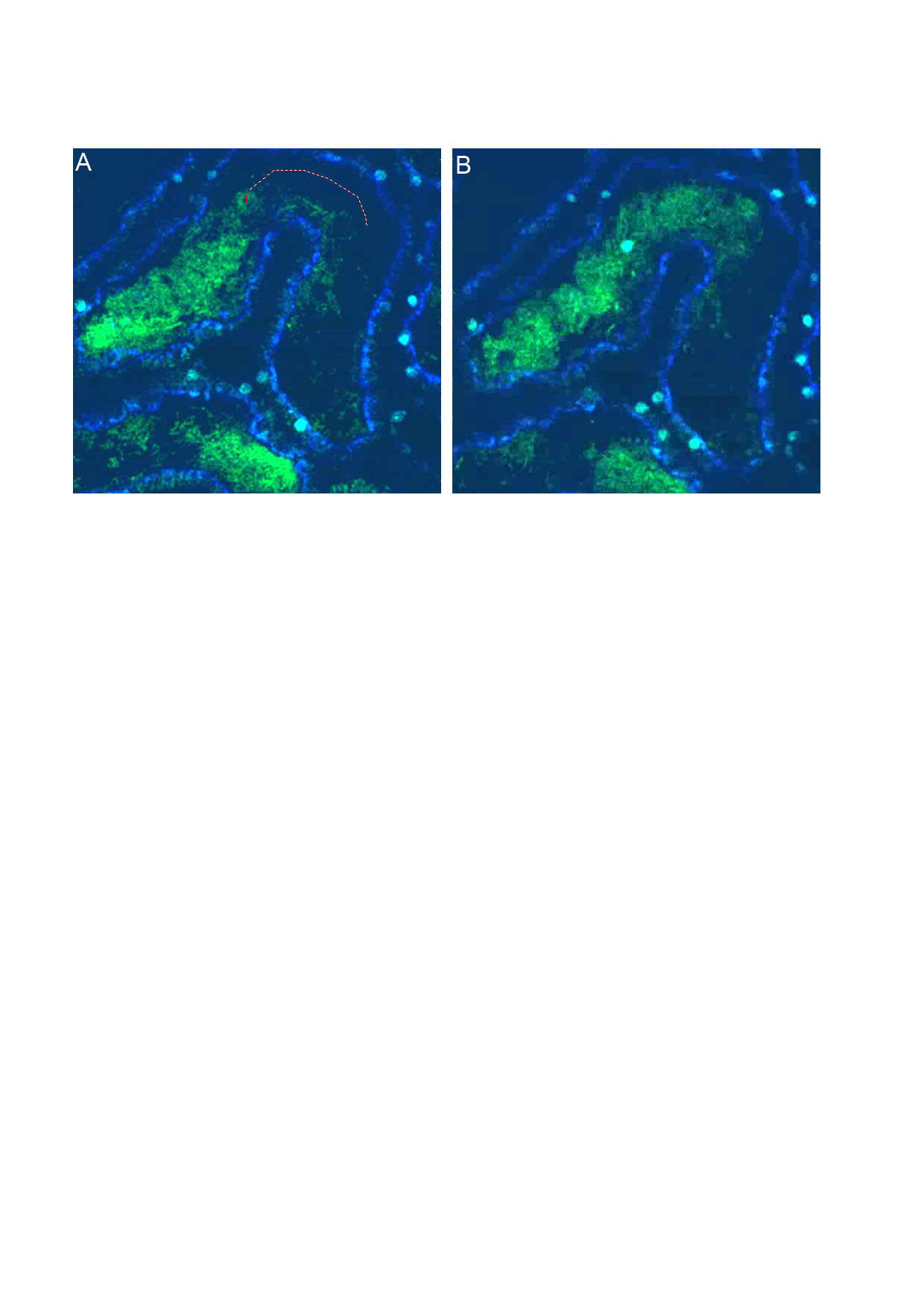

Supplement: Figure S1 — Movement of ARD42 through a proximal tubule. (A) Image taken at the beginning of the video showing the position of the bacteria and a trace line showing the path they travel over the 70 s duration. (B) Image taken at the end of the video, 70 s later, showing bacterial position. (0.75 MB TIF) [file ppat.1001298.s001.tif]
